# Editorial: Strengthening Health System and Community Responses to Confront COVID-19 Pandemic in Resource-Scare Settings

**DOI:** 10.3389/fpubh.2022.935490

**Published:** 2022-07-06

**Authors:** Bach Xuan Tran, Linh My Tran, Jongnam Hwang, Hoa Do, Roger Ho

**Affiliations:** ^1^Institute for Preventive Medicine and Public Health, Hanoi Medical University, Hanoi, Vietnam; ^2^Institute of Health Economics and Technology (iHEAT), Hanoi, Vietnam; ^3^Division of Social Welfare & Health Administration, Wonkwang University, Iksan, South Korea; ^4^Center of Excellence in Public Health Nutrition, Nguyen Tat Thanh University, Hanoi, Vietnam; ^5^Department of Psychological Medicine, National University of Singapore, Singapore, Singapore

**Keywords:** health systems, COVID-19, capacity, responses, community, LMIC

The COVID-19 pandemic has substantially affected global communities and health systems in both high- and low-income countries. Many nations have been experiencing very high population burdens that implies the importance of strengthening responses systems and mobilizing communities in pandemic preparedness and control. The shortage of resources in LMICs is well-documented prior to COVID-19 and it has been worsened with the surge of COVID-19. In many LMICs, testing capacity remains inadequate, the number of ICU beds is far less than required, access to drug treatments such as dexamethasone is limited, and the supply of therapeutic oxygen and ventilators is insufficient (1). The scarcity is caused by various factors including the migration to developed countries, shortage of supplies, poor healthcare infrastructure, limited ICU facilities, and lack of access to guidelines and protocols (2). Important difficulties that LMICs are facing are the insufficient testing capacity and the shortage of healthcare providers, which precluded accurate assessment of disease burden and subsequently resource allocations (2). This Research Topic summarizes experience of low- and middle-income countries (LMICs) in managing both the epidemiological transition and the threat of emerging infectious diseases. It offers the ideal opportunity at this critical juncture in the development of global health to identify key lessons for health system strengthening in LMICs.

The following studies summarized experience of LMICs in managing the surge of COVID-19 in terms of capacities and responsiveness of health systems and communities. Zhang studied the experience of public health system construction in China's COVID-19 prevention and identified key lessons for other countries to confront unprecedented pandemic. The paper not only pointed out several advantages of China's public health system construction in response to COVID-19, such as adequate supply of health resources and improved affordability of health care, but also discussed China's deficiencies, including low utilization efficiency of health resources, unequal ability to pay for medical expenses, and late disclosure of virus information in the early stage of the outbreak of COVID-19. Given that Nepal is facing a flood of COVID-19 cases after the lockdown was lifted in July 2020, Rayamajhee et al. provided critical insights on response of the government, highlighting the need to increase testing, tracing, and isolation capacity, and to set up quality quarantine centers throughout the nation to address the rise in COVID-19 infection cases. As cases have continued to fluctuate over a year into the pandemic in Indonesia, Mahendradhata et al. reviewed the current capacity of the healthcare system to respond to COVID-19 and emphasized the need for the Indonesian Government to ramp up the country's healthcare capacity in order to absorb and accommodate the varying healthcare demands during the pandemic. Saha and Gulshan provided a systematic analysis of the overall patterns in terms of number of cases, number of deaths, and impacts of COVID-19 in Bangladesh. The authors also shed light on the underlying causes that resulted in a continuous outbreak while discussing possible measures, effectiveness of the preparedness, implementation gaps, and their consequences to gather vital information and prevent future pandemics. Abd El Ghaffar et al. investigated the situation in some of the COVID-19 screening hospitals in Egypt in terms of inpatient beds, ICU beds, and ventilator utilization rates. Results from this paper, which indicated a shortage of resources, would help policymakers make informed resource reallocation decisions in Egypt, or other developing countries, which suffered from a lack of resources and a weak health system prior to the pandemic. Coumare et al. discussed the challenges and experiences in preparation and responses to the ongoing COVID-19 pandemic at a tertiary teaching hospital situated at Puducherry, India. Tran et al. examined factors associated with the intention to participate in COVID-19 frontline prevention activities among Vietnamese nursing students. The finding suggested that socioeconomic characteristics of participants, their source of COVID-19 related knowledge, and their perception and attitude toward participating in COVID-19 frontline activities were associated with intention to participate. Given resource-scarce settings of most LMICs, these countries are challenged by their limited capacity for manufacturing test kits, limited budgets for equipment and reagents, and scant ability to make competitive bids for global supplies. Gyawali and Mohammad Al-Amin et al. advocated for recognition of the almost insurmountable obstacles that face the implementation of non-pharmaceutical interventions for COVID-19 management in Asian LMICs and called for a global commitment to the equitable distribution of therapeutants and vaccines. Although the World Bank is making available more than $160 billion funding to LMICs to purchase and distribute COVID-19 vaccines, tests and treatment and mitigate the health, economic, and social shocks, and philanthropic organizations such as the Global Alliance for Vaccines (GAVI) have received billions of dollars in donations, much more will be required for LMICs to combat COVID-19 and mitigate the expected economic downturn. In the long term, LMICs will need to develop their own capacity to manufacture and distribute vaccines, as well as strengthen primary health centers to manage the impact of the pandemic.

In addition to capacities and responsiveness of health systems and communities to respond to the pandemic, numerous studies have highlighted the importance of strategic planning and resource mobilization to maintain essential health services and maximize human resource capacity to address COVID-19 situation and its associated disruptions. Muhammad Nur Amir et al.. described the workforce mobilization from the National Institutes of Health (NIH) to the other public healthcare facilities within the Ministry of Health (MOH) in the early phases of the COVID-19 pandemic management in Malaysia. The paper demonstrated how this workforce mobilization team efficiently mobilize the healthcare workforce and fulfill requests received for human resource aid, resulting in reduced infected COVID-19 cases throughout the country. Nguyen D. N. et al. provided evidence for reforming the training programs to prepare medical students, a potential task force with the capability to support the stretched health sector, for COVID-19 responses in Vietnam. This study suggested that the training curriculum should include both theoretical approaches (e.g., pathology and critical treatments) as well as other contextual approaches to achieve efficient epidemic control in specific regions. Le et al. illustrated the potential and feasibility of intersectoral collaboration in epidemic preparedness and response at grassroots levels in the threat of COVID-19 pandemic in Vietnam, which ensures sufficient resources for urgent cases and helps to inform a determined response framework.

Given the speed and spread of transmission of COVID-19, assessing the effectiveness of epidemiological monitoring and surveillance of the epidemics is critical to control the expansion of the epidemics and avoid the collapse of health care systems. Shewade et al. reported that the estimated COVID-19 deaths in India after adjusting for the coverage and quality of the routine death surveillance may be 5.5–11 times the reported COVID-19 deaths. Hence, this study further discussed the routine deaths surveillance in India, the rationale for adjusting the reported COVID-19 deaths for coverage of the routine death surveillance and its implications on the estimated infection fatality ratio (IFR) for India in order to make meaningful and reliable comparisons. Mansab et al. highlighted the disparity in oxygen provision for COVID-19 patients between 26 nations included in the study and suggested that there was an association with higher national mortality rates in those nations that pursued a conservative oxygen strategy. Abbasi-Kangevari et al. sought to determine the priorities of the Iranian public toward the fair allocation of ventilators during the COVID-19 pandemic. Participants stated that socioeconomic factors, except for age > 80, should not be involved in prioritizing mechanical ventilators at the time of resources scarcity. Front-line physicians and nurses of COVID-19 patients, pregnant mothers, mothers who had children under 2 years old had been given high priority.

Many articles in this Topic focused on evaluating the effectiveness of epidemiological, psychosocial and economical interventions to prevent the importation, spreading and relieve the impact of COVID-19 pandemic in LMICs. Vietnam has achieved initial results in flattening the curve and slowing the spread of COVID-19 transmission in the community, which is attributable to a high-level adherence with social distancing measures, accompanied with contact tracing, mass testing, and mandatory isolation. Vo et al. demonstrated how social distancing measure had been implemented in Vietnam and further discussed factors associated with the high compliance of Vietnamese with social distancing measures. Tam et al. discussed the evidence on the effectiveness, and rationale for community mass masking to prevent the COVID-19 transmission in Vietnam. Sun et al. showed that the proper management of inbound travelers from outbreak areas had a significantly positive effect on the prevention and control of the virus and suggested that effective measures taken by Yunnan province may provide an important reference for preventing the COVID-19 outbreak in other regions. As the prevalence of psychological stress among healthcare workers (HCWs) in Vietnam during the COVID-19 pandemic was high, Nguyen P. T. L. et al. sought to understand COVID-19-related, psychological stress risk factors among HCWs, their concerns and demands for mental health support during the pandemic period. The author recommended psychological interventions involving web-based consulting services to provide mental health support among HCWs. Nguyen A. N. et al. assessed the knowledge and practices regarding the prevention of the COVID-19 among the HCWs in Vietnam to identify the ways of disseminating information to maximize the safety of these essential workers. Findings from this study suggested that future education initiatives should centre initially on the COVID-19 virus aerosols with the primary focus on doctors, especially those in emergency and the intensive care departments. In addition to epidemiological and psychosocial interventions, Nguyen H. T. T. et al. described economic recovery solutions implemented by the Vietnam government to manage the fiscal deficit, such as focus on effectively implementing domestic stimulus, use the savings from falling international oil prices to curb the crisis, earn funding from the World Bank WB and the International Monetary Fund, etc.

Several studies have raised important points regarding the impact of perceptions and attitudes toward the COVID-19 crisis on the improvement of both public health and individual well-being and highlighted how digital health care and social media may influence public's attitude during the COVID-19 outbreak. Specifically, Klement pointed out that journalism, politics, and medicine involved within the COVID-19 crisis have maintained a simple narrative and reductionist thinking and emphasized the need for systems thinking during the outbreak. A survey by Raza et al. was conducted to understand knowledge, attitudes, and practices related to COVID-19 among the students in Lahore, Pakistan. This survey showed that most of the students were well informed about COVID-19 and exhibited a proactive approach during the outbreak, suggesting an effective public health campaign of the local government to deliver public health knowledge in the community. Chatterjee et al. focused on highlighting some of the key aspects of digital healthcare during the times of COVID-19 pandemic in South America. This study shed light on the role of Artificial Intelligence and the Internet of Things role in resource optimization along their potential applications like clinical decision support systems and predictive risk modeling, especially in the direction of combating the emergent challenges due to the COVID-19 pandemic. Mat Dawi et al. examined the influence of e-government and social media on the public's attitude to adopt protective behavior and suggested that during the COVID-19 outbreak, public health decision makers may use e-government and social media platforms as effective tools to improve public engagement on protective behavior. Although Nguyen, Nguyen, Le, et al. demonstrated a high level of agreement among the general population toward the importance and necessity of national response measures to combat the COVID-19 epidemic in Vietnam, Nguyen, Nguyen, Nguyen, et al. expressed concern about the impact of fake news on the adherence of national response measures during the lockdown period in Vietnam. In response to fake news, the government had made early predictions and concrete strategies, such as passing a cybersecurity law or establishing official communication channels, which may also have important lessons for other nations in the fight against COVID-19.

Besides efforts to mitigate pandemics in LMICs from medical staff training and monitoring to system modernization, it is also important to consider potential barriers that arise during the COVID-19 vaccine rollout in LMICs and how to address these difficulties in resource-scarce settings. Recent studies have reported several challenges for vaccination faced by LMICs, including vaccine hesitancy (3), inadequate cold-chain and storage (3), low resource availability (4), poor roads to transport vaccines (5), lack of coordination (7), and limited funds for surveillance (6, 7). These issues could be partially addressed from strategies discussed in this topic, such as expanding healthcare workforce capacity to increase vaccination speed, and improving the population willingness to vaccinate. For example, several articles have described strategies to maximize human resource capacity to address COVID-19 situation and pointed out the need to improve healthcare capacity in order to accommodate the varying healthcare demands during the pandemic (Gyawali and Mohammad Al-Amin et al.; Mahendradhata et al.; Rayamajhee et al.; Tran et al.; Zhang). Ensuring sufficient healthcare workforce, training more health providers, and recruiting volunteers would help to increase vaccine availability, distribution, and monitoring. In addition to healthcare workforce capacity, vaccine acceptance and hesitancy remain a significant barrier to increase vaccination rate. Perceptions and attitudes play an important role on the improvement of both public health and individual well-being, which significantly influenced by Internet and social media (Chatterjee et al.; Klement; Mat Dawi et al.; Nguyen, Nguyen, Le, et al.; Nguyen, Nguyen, Nguyen, et al.; Raza et al.). Thus, governments could establish awareness-building initiatives using community and mass media to manage inaccurate information about vaccines, lack of information, and lack of trust in the government and pharmaceutical companies. Lastly, governments should consider to increase budgets for COVID-19 vaccine purchase and delivery to scale up COVID-19 vaccination and offer access to all populations; however, this has not been discussed in the collection of papers in this topic.

The COVID-19 pandemic has challenged the healthcare capacity of many countries. The collection of papers in this topic provides insight on the opportunities and challenges in strengthening health system and community responses to confront COVID-19 pandemic in resource-scarce settings. Lessons learned in terms of strength and limitations from the latest evidence on health sectors and community responses to control the COVID-19 pandemic in LMICs would suggest implications to improve the effectiveness of current policies and facilitate development of new strategies to strengthen the capacity and efficiency of healthcare systems in the fight against COVID-19. Governments will continue to play an important role in responding to the coronavirus pandemic and providing access to care for communities in resource-constrained countries with limited human, infrastructural, and financial resources and that are behind the curve in the spread of the pandemic.

## Author Contributions

All authors listed have made a substantial, direct, and intellectual contribution to the work and approved it for publication.

## Funding

Research was supported by the Vingroup Innovation Foundation (VINIF) in project code VINIF.2020.Covid-19.DA03. The article processing charge for journal is financed by NUS Department of Psychological Medicine (R-177-000-100-001/R-177-000-003-001/R177000702733) and NUS iHeathtech Other Operating Expenses (R-722-000-004-731).

## Conflict of Interest

The authors declare that the research was conducted in the absence of any commercial or financial relationships that could be construed as a potential conflict of interest.

## Publisher's Note

All claims expressed in this article are solely those of the authors and do not necessarily represent those of their affiliated organizations, or those of the publisher, the editors and the reviewers. Any product that may be evaluated in this article, or claim that may be made by its manufacturer, is not guaranteed or endorsed by the publisher.
